# Deriving a Simple Clinical Predictive Score for Posterior Circulation Ischemic Stroke (PCS-SCORE)

**DOI:** 10.1177/11795735261424050

**Published:** 2026-02-12

**Authors:** Yahia Imam, Rajvir Singh, Prem Chandra, Ishrat Hakeem, Saadat Kamran, Ahmad Muhammad, Salman Al Jerdi, Suhail Hussain, Khawaja Hassan Haroon, Jon D. Perkins, Ahmed Elsotouhy, Mohamed Sayed Abdelmoneim, Zain A. Bhutta, Mostafa Mahmoud, Ehab Mahmoud, Osman Koc, Dirk Deleu

**Affiliations:** 1 Neuroscience Institute, 36977 Hamad Medical Corporation, Doha, Qatar; 2 36579 Department of Medical education, Weill Cornell Medicine-Qatar, Doha, Qatar; 3 College of Medicine, Qatar University, Doha, Qatar; 4 Cardiology Research Center, 36977 Hamad Medical Corporation, Doha, Qatar; 5 Statistics, Medical Research Center, 36977 Hamad Medical Corporation, Doha, Qatar; 6 Department of Emergency Medicine, 36977 Hamad Medical Corporation, Doha, Qatar; 7 Department of Emergency Medicine and Services, Helsinki University Hospital, University of Helsinki, Helsinki, Finland

**Keywords:** altered mental status, central nervous system, coma

## Abstract

**Background:**

Posterior circulation ischemic stroke (PCS) accounts for up to 25% of all ischemic strokes but remains frequently under-recognized due to atypical symptoms and poor representation in conventional stroke scales. Early diagnosis is critical yet challenging. This study aimed to derive a pragmatic clinical scoring tool, the PCS-SCORE, to identify patients at high risk of PCS based solely on bedside features.

**Methods:**

We retrospectively analyzed 5163 patients from a prospective stroke registry, including 1571 with -confirmed PCS. Key predictors were identified through multivariable logistic regression and lasso modeling. Variables were weighted according to regression coefficients and clinical relevance. The final PCS-SCORE (0-9 points) included: diabetes (1 point), hypertension (1), male sex (1), double/blurred vision (2), vertigo with vomiting (2), and incoordination (2).

**Results:**

At a score threshold >3, the PCS-SCORE achieved an area under the curve (AUC) of 0.76, with 87.9% specificity and 43.4% sensitivity. Raising the threshold to >4 increased specificity to 94.4% (sensitivity 27.9%). Higher scores corresponded with progressively increased likelihood of PCS, enabling confident identification of high-risk patients.

**Conclusion:**

The PCS-SCORE is a simple, highly specific bedside tool for early detection of posterior circulation strokes. Its rule-in strength makes it especially useful in prehospital settings, resource-limited environments, and crowded emergency departments. Prospective validation is ongoing.

## Plain Language Summary

Posterior circulation ischemic stroke (PCS) represents up to a quarter of all ischemic strokes but is often missed because symptoms can be atypical and current stroke scales under-represent these cases. Prompt recognition is crucial for timely treatment, yet diagnosis remains challenging, particularly in prehospital or resource-limited settings. This study set out to develop a practical, easy-to-use clinical scoring tool—the PCS-SCORE—designed to identify patients at high risk of PCS based entirely on bedside findings. Researchers retrospectively reviewed data from a prospective stroke registry containing 5,163 patients, of whom 1,571 had confirmed PCS. Predictive factors were identified using multivariable logistic regression and lasso modeling. Each factor was assigned a point value based on statistical weight and clinical significance. The final PCS-SCORE ranges from 0 to 9 points and includes six predictors: diabetes mellitus (1 point), hypertension (1 point), male sex (1 point), double or blurred vision (2 points), vertigo with vomiting (2 points), and incoordination (2 points). Performance analysis showed that at a threshold score greater than 3, the PCS-SCORE achieved an area under the curve (AUC) of 0.76, with 87.9% specificity and 43.4% sensitivity. Increasing the threshold to greater than 4 improved specificity to 94.4%, though sensitivity decreased to 27.9%. Importantly, higher PCS-SCORE values correlated with a markedly increased probability of PCS, supporting its utility as a “rule-in” diagnostic aid. It’s high specificity makes it particularly useful for quickly identifying likely PCS cases, helping clinicians prioritize further imaging and urgent intervention. This is especially valuable in crowded emergency departments, rural or low-resource hospitals, and prehospital environments where rapid triage decisions are critical. In conclusion, the PCS-SCORE is a simple, bedside clinical tool with strong potential to improve early detect.

## Introduction

Posterior circulation ischemic stroke (PCS) comprises up to a quarter of all ischemic strokes.^1^ They are often elusive to diagnose due to nonspecific symptoms and the poor performance of commonly used clinical screening tools, such as F.A.S.T,^2^ the Recognition of Stroke in the Emergency Room (ROSIER) Scale.^3^ and the National Institutes of Health Stroke Scale (NIHSS),^4^ which are predominantly designed to detect anterior circulation events.^5^

Additionally, standard computed tomography (CT), the most widely available neuroimaging modality in emergency departments (EDs), often fails to detect PCS, leading to missed diagnoses and lost therapeutic opportunities.^6^ This diagnostic ambiguity contributes to physician anxiety, particularly in patients presenting with dizziness, and has driven an over-reliance on advanced imaging techniques such as CT perfusion and MRI.^7^ While more sensitive, these tools increase emergency room wait times, lead to potentially avoidable admissions, inflate bed occupancy, and consume valuable healthcare resources.^8-10^

Several attempts have been made to improve bedside diagnosis of PCS by adapting existing tools,such as BE-FAST (Balance, Eyes, Face, Arm, Speech, Time)^11^ and POST-NIHSS,^5^ or by introducing PCS-specific scales like the Adam’s Scale of Posterior Stroke (ASPOS),^12^ the Israeli Vertebrobasilar Stroke Scale (IVBSS),^13^ the TriAGe + Score,^14^ and the PCI score.^15^ While these instruments show promise, most were developed from relatively small cohorts, exhibit varying levels of complexity, and remain underutilized in real-world clinical practice.

Given the high morbidity associated with missed or delayed PCS diagnosis — particularly in strokes affecting the brainstem or cerebellum — there is a pressing need for a simple, bedside tool that enables clinicians to estimate the likelihood of PCS based on clinical presentation. Such a tool must be pragmatic, intuitive, and scalable across settings, from rural EDs to high-volume urban stroke centers.

In this study, we leveraged a large, prospectively maintained stroke database enriched with MRI-confirmed diagnoses to derive a weighted clinical score based entirely on historical and physical exam findings. Our goal was to create a rule-in tool, the PCS-SCORE, that facilitates early detection of posterior strokes and prioritizes high-risk patients for urgent neuroimaging and intervention.

## Methods

This was a retrospective derivation study using prospectively collected registry data, intended for score development and internal validation from the Qatar Stroke Database spanning 2014-2023. The registry includes all patients presenting with suspected acute stroke to Hamad General Hospital, the principal stroke center in Qatar.

Inclusion and exclusion criteria:

We included PCS patient fulfilling these criteria as published previously^1^:

Acute neurological deficit attributable to posterior circulation ischemia, including basilar artery occlusion, confirmed by computed tomographic angiography (CTA), magnetic resonance angiography (MRA), or conventional cerebral angiography.

Acute neurological deficit with brainstem involvement, manifesting as alternating hemiplegia or tetraplegia, brainstem signs, visual loss, locked-in state, coma, or death.

Coma or loss of consciousness associated with acute posterior circulation infarction demonstrated on CT or MRI.

Accordingly, PCS was defined radiologically as ischemic infarction involving the brainstem, cerebellum, occipital lobes, and/or thalami.

The PCS cohort was compared with other patients in the registry, most of whom were initially suspected of having posterior circulation stroke at first presentation but were subsequently diagnosed with stroke mimics (eg, peripheral vestibular disorders), other medical conditions, or anterior circulation ischemic stroke following definitive evaluation.

Patients were excluded only if key clinical variables required for score derivation were missing or if intracerebral hemorrhage was present.

Demographic data (age, sex), vascular risk factors (pre-existing, physician-diagnosed hypertension, diabetes mellitus), and presenting symptoms (vertigo, vomiting, diplopia, blurred vision, dysarthria, incoordination, limb weakness) were extracted. Each case underwent independent adjudication by stroke neurologists and neuroradiologists.

Univariate and multivariate logistic regression models identified variables significantly associated with PCS. The final scoring model used coefficients from the multivariable model weighted and rounded to integers. Variables were assigned 1 or 2 points based on clinical relevance and statistical strength. The maximum score was 9.

Statistical methods included logistic regression (stepwise backward) and lasso regression (λ minimized via cross-validation). Model discrimination was evaluated using AUC. Diagnostic indices, sensitivity, specificity, PPV, and NPV were calculated at several score thresholds.

## Results

Out of 5163 patients, 1571 had MRI-confirmed PCS. The six predictors retained in the PCS-SCORE were: diabetes mellitus (1 point), hypertension (1), male sex (1), double/blurred vision (2), vertigo with vomiting (2), and incoordination (2) (Table 1).

**Table 1.** Scoring System

Predictors	Regression coefficient (β)	Exact Scores	Weighted scores (rounded)
Diabetes Miletus	0.814	1	1
Hypertension	0.924	1.13	1
Double/blurred vision	1.354	1.67	2
Vertigo with vomiting	1.157	1.42	2
Sex	0.953	1.17	1
Coordination	1.108	1.36	2

**Figure 1.**
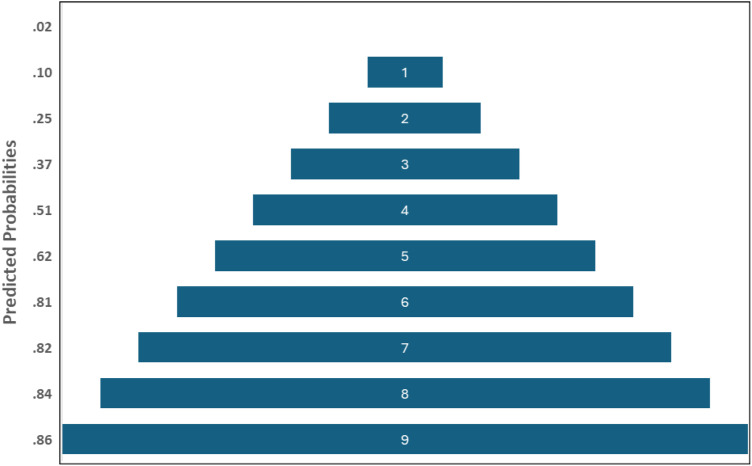
Predicted probability of PCS with varying weighted score values

At a cutoff >3 (Table 2), the PCS-SCORE yielded a sensitivity of 43.67% and specificity of 87.86%, with a positive likelihood ratio of 3.60, negative likelihood ratio of 0.64, PPV of 61.14%, and NPV of 78.10%. Increasing the threshold to >4 (Table 3) improved specificity to 94.38% and PPV to 68.44%, but reduced sensitivity to 27.88%, with a positive likelihood ratio of 4.96. The predictive probability of PCS rose steadily from 6% (score = 1) to 94% (score = 9) Figure 1.

**Table 2.** Diagnostic Performance of PCS-SCORE at Cutoff >3

Parameter	Estimate	95% CI
Sensitivity	43.67%	41.23%-46.13%
Specificity	87.86%	86.75%-88.89%
PPV	61.14%	58.26%-63.95%
NPV	78.10%	76.80%-79.35%
Diagnostic accuracy	74.41%	73.21%-75.59%
Positive likelihood ratio	3.597	3.568-3.627
Negative likelihood ratio	0.6412	0.6397-0.6426
Diagnostic odds ratio	5.611	4.871-6.463
Cohen’s Kappa	0.3428	0.3163-0.3694

**Table 3.** Diagnostic Performance of PCS-SCORE at Cutoff >4

Parameter	Estimate	95% CI
Sensitivity	27.88%	25.72%-30.15%
Specificity	94.38%	93.57%-95.08%
PPV	68.44%	64.74%-71.92%
NPV	74.95%	73.67%-76.19%
Diagnostic accuracy	74.14%	72.93%-75.32%
Positive likelihood ratio	4.958	4.853-5.064
Negative likelihood ratio	0.7642	0.7628-0.7655
Diagnostic odds ratio	6.488	5.42-7.765
Cohen’s Kappa	0.2671	0.2437-0.2905

**Figure 2.**
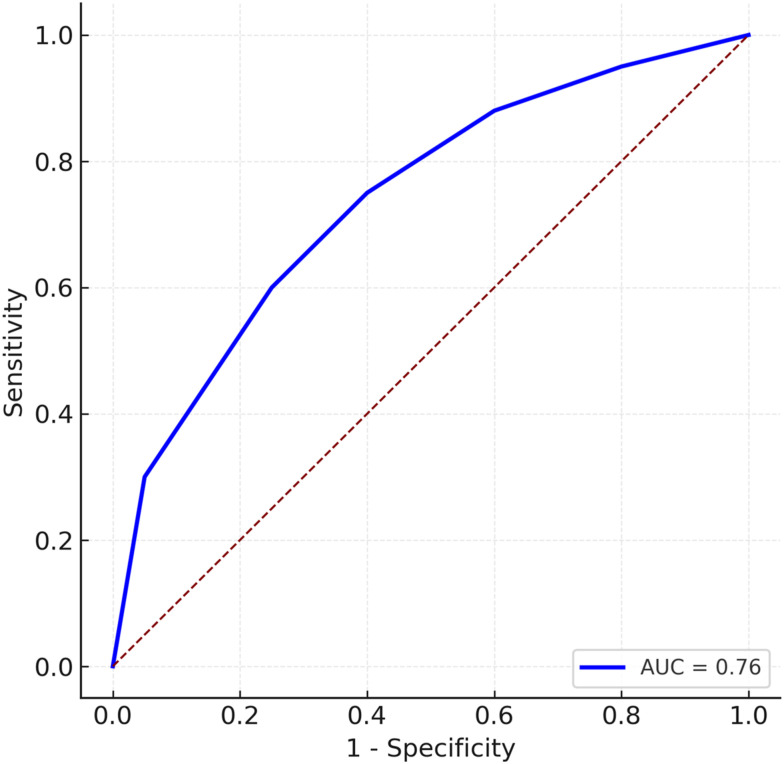
Receiver Operating Characteristic (ROC) curve for the PCS-SCORE predicting posterior circulation ischemic stroke (PCS): The area under the curve (AUC) was 0.76, indicating good discrimination between PCS and non-PCS cases. The blue curve represents the performance of the PCS-SCORE model, while the diagonal reference line indicates chance-level discrimination

Receiver operating characteristic (ROC) analysis demonstrated an area under the curve (AUC) of 0.76, indicating good overall discrimination between PCS and non-PCS cases. As illustrated in Figure 2, higher score thresholds improve specificity at the cost of sensitivity, reinforcing the PCS-SCORE’s utility as a high-specificity, rule-in tool for identifying patients who warrant urgent imaging and intervention.

## Discussion

Dizziness accounts for nearly 4 million ED visits annually in the U.S., yet posterior circulation stroke is often overlooked in this population due to its non-specific presentation and lack of focal signs.^16^ The PCS-SCORE addresses this gap by offering a pragmatic rule-in tool tailored to bedside evaluation, helping to prioritize imaging in high-risk presentations.

This study introduces the PCS-SCORE; a pragmatic, bedside clinical tool designed to improve early identification of posterior circulation stroke (PCS) using binary clinical features alone. Unlike traditional scales such as FAST, ROSIER, or the NIH Stroke Scale (NIHSS), which predominantly emphasize anterior circulation symptoms like aphasia and hemiparesis, the PCS-SCORE was intentionally developed to capture the more subtle and non-localizing signs typical of PCS, including vertigo, diplopia, and incoordination.^17^ These atypical symptoms, often mimicked by benign conditions such as vestibular neuritis or migraine, contribute to frequent misclassification and delayed diagnosis.^16^

Although extended tools like BEFAST and POST-NIHSS have attempted to address these limitations, they either add complexity or still rely on structured neurological assessments not easily applied in fast-paced emergency settings. Similarly, posterior-specific scales such as the Adam’s Scale of Posterior Stroke (ASPOS), Israeli Vertebrobasilar Stroke Scale (IVBSS), PCI Score, and TriAGe+, while conceptually valuable, are either derived from small cohorts, require neuroanatomical interpretation, or are heavily dependent on advanced imaging, limiting their generalizability.

The PCS-SCORE distinguishes itself in several keyways. It is derived from over 5000 registry-confirmed stroke cases and internally validated against final MRI-based diagnoses. It integrates six high-yield features (each weighted 1 or 2 points) identified through multivariable regression, culminating in a total score out of 9. At thresholds above 4, the tool demonstrates specificity exceeding 94%, enabling clinicians to confidently rule in PCS in high-risk patients. Table 4.

**Table 4.** Comparison Between Different Posterior Circulation Stroke Scales

Scale	Components	Complexity	Derivation cohort	Validation	Key limitations	How PCS-SCORE is different
ASPOS (Adam’s Scale of Posterior Stroke)	7 clinical signs including nausea, vomiting, and ataxia	Moderate (requires structured neuro exam)	102 patients, China	Internal only	Small cohort, unfamiliar internationally	PCS-SCORE is simpler, uses binary history items, and based on >5000 patients
POST-NIHSS	Adds posterior signs to NIHSS	High (adds to NIHSS administration time)	Conceptual framework	Limited	NIHSS remains anterior-heavy, requires trained raters	PCS-SCORE stands alone without modifying NIHSS; easier for EDs/prehospital
IVBSS (Israeli Vertebrobasilar Stroke Scale)	7 posterior signs including eye movement and cranial nerve deficits	Moderate to high	Small single-center (Israel)	No broad validation	Complexity, neuroanatomy knowledge required	PCS-SCORE only needs yes/no symptom responses; practical for all levels
PCI score	Mix of clinical and imaging findings	High (requires MRI input)	Small sample, neuroimaging-based	Conceptually sound	Requires imaging for scoring	PCS-SCORE is fully imaging-independent — designed for early bedside use
TriAGe+	Clinical score for dizziness/vertigo	Moderate (vertigo-focused)	396 patients	Internally validated	Not stroke-specific; misses non-dizzy PCS	PCS-SCORE is explicitly developed for posterior circulation stroke, not vestibular disorders
PCS-SCORE (this study)	6 variables: DM (1), HTN (1), male sex (1), double/blurred vision (2), vertigo with vomiting (2), incoordination (2)	Low – fast bedside score (0-9)	5163 patients from multiethnic registry, 1571 PCS confirmed by MRI	Ongoing prospective validation	Prioritizes rule-in, may miss mild cases (modest sensitivity)	Balanced, weighted scoring, strong specificity (>94%), easy to apply at bedside or triage

Male sex remained an independent predictor in both multivariable logistic and lasso regression models after adjustment for age and vascular comorbidities, reflecting a probably observed epidemiological association rather than a proposed biological mechanism. Nevertheless, several sex specific studies have shown a male predilection to posterior circulation stroke.^18,19^

And while vertigo with vomiting are non-specific and may point to either central or peripheral etiology when considered in isolation. Its inclusion in the PCS-SCORE reflects its incremental value only when combined with other posterior circulation features and vascular risk factors, lending it more weight and mitigating its diagnostic overlap with peripheral vestibular disorders.

This specificity is not merely a statistical artifact as it has tangible clinical utility. In emergency departments (EDs) operating under strain, low-specificity tools can flood diagnostic pathways with false positives, driving unnecessary imaging, prolonged wait times, bed overuse, and increased system inefficiency.^7,10^ Crucially, they also contribute to provider mental fatigue (a state of cognitive overload stemming from sustained diagnostic uncertainty, repetitive decision-making, and pressure to avoid missed diagnoses).^20^ This is particularly problematic in PCS, where vague symptoms like dizziness, nausea, or unsteadiness often lack clear localizing signs. Clinicians, faced with ambiguous presentations, may default to defensive practice patterns: over-reliance on MRI, unnecessary admissions, or low-threshold referrals, all of which inflate workload without improving yield.

The PCS-SCORE is not intended to determine whether neuroimaging should be performed nor to delay reperfusion therapy. All patients with suspected stroke should continue to follow established acute stroke pathways. Rather, the score functions as a triage-support, rule-in tool, assisting clinicians in prioritizing high-probability PCS cases for urgent imaging in settings with diagnostic congestion or limited resources.

This approach enhances diagnostic precision by providing clinicians with a potential cognitive scaffold to mitigate alarm fatigue and the resultant downstream over-investigation.^21,22^ While high sensitivity is desirable for broad screening tools, the PCS-SCORE was not intentionally designed as a high-specificity instrument a priori. Rather, high specificity emerged empirically during model derivation and performance analysis. This observed rule-in strength suggests that the PCS-SCORE may complement existing high-sensitivity screening tools, such as FAST or BE-FAST, by helping prioritize patients with a higher probability of posterior circulation stroke in selected clinical settings thus, filling a critical gap in posterior circulation stroke triage where specificity, simplicity, and real-world usability converge.

### Limitation

Limitations include retrospective derivation, absence of external prospective validation at this stage, and reliance on subjective symptom reporting (eg, vertigo, incoordination). As a registry-based derivation study, a formal a priori sample size calculation was not performed; however, the large cohort size (>5000 patients) exceeds that of most previously published posterior circulation scoring systems.

## Conclusion

The PCS-SCORE is a simple, clinically intuitive tool that can support early diagnosis of posterior circulation strokes. It confidently identifies high-risk patients and streamlines imaging and treatment pathways, Prospective validation in real-life scenarios is needed.

## Data Availability

The datasets generated and/or analyzed during the current study are not publicly available due to institutional data protection policies but are available from the corresponding author on reasonable request and with appropriate ethical approvals.*
